# Adrenal Function in Adolescence is Related to Intrauterine and Postnatal Growth

**DOI:** 10.3390/medicina55050167

**Published:** 2019-05-20

**Authors:** Indrė Petraitienė, Margarita Valūnienė, Kerstin Albertsson-Wikland, Rasa Verkauskienė

**Affiliations:** 1 Department of Endocrinology, Medical Academy, Lithuanian University of Health Sciences, 44307 Kaunas, Lithuania; rasa.verkauskiene@gmail.com; 2 Mother and Child’s Clinic, Republican Siauliai County Hospital, 76231 Siauliai, Lithuania; valuniene@gmail.com; 3 Department of Physiology/Endocrinology, Institute of Neuroscience and Physiology, The Sahlgrenska Academy, University of Gothenburg, 405 30 Gothenburg, Sweden; kerstin.albertsson.wikland@gu.se; 4 Institute of Endocrinology, Medical Academy, Lithuanian University of Health Sciences, 44307 Kaunas, Lithuania

**Keywords:** children born appropriate for gestational age (AGA), DHEAS, cortisol, puberty, postnatal growth

## Abstract

*Background and objectives:* Intrauterine growth restriction is thought to be implicated in long-term programming of hypothalamic–pituitary–adrenal axis activity. We investigated adrenal function in adolescents born small for gestational age (SGA) in relation to their postnatal growth and cardiovascular parameters. *Materials and Methods:* Anthropometric parameters, blood pressure, heart rate, dehydroepiandrosterone sulfate (DHEAS), and cortisol levels were assessed in 102 adolescents aged 11–14 years followed from birth (47 SGA and 55 born appropriate for gestational age (AGA)). *Results:* Mean DHEAS levels were higher in SGA adolescents with catch-up growth (SGA_CU+_), compared with AGA. Second-year height velocity and body mass index (BMI) gain during preschool years were positively related to DHEAS levels. Morning cortisol levels and systolic and diastolic blood pressure were higher in SGA adolescents without catch-up growth (SGA_CU−_) compared with AGA. Second-year BMI gain was inversely, and 2–12 years increase in subscapular skinfold thickness was directly associated with cortisol levels. Size at birth and postnatal growth explained 47.8% and 38.2% of variation in DHEAS and cortisol levels, respectively. *Conclusion:* Adrenal function in adolescence is affected by prenatal and postnatal growth: small size at birth with postnatal catch-up growth is related to higher DHEAS secretion, whereas increased cortisol levels and blood pressure are higher in short SGA adolescents.

## 1. Introduction

Growth in utero may have implications for growth and development throughout life. Children born small for gestational age (SGA) have been shown to be at higher risk of metabolic consequences later in life, such as obesity, insulin resistance, dyslipidemia, and hypertension [1,2]. It has been suggested that these effects may arise owing to fetal programming of the hypothalamic–pituitary–adrenal (HPA) axis [1]. Stressful conditions in utero may lead to small birth size, alterations in regulation of the HPA axis and increased cortisol levels in both mothers and infants [3,4]. Cortisol plays a central role in the regulation of human metabolism [5], and disturbed secretion of cortisol may in turn be related to increased risk of metabolic syndrome [2,6,7]. In addition, activation of the HPA axis may be demonstrated by exaggerated adrenarche and increased dehydroepiandrosterone sulfate (DHEAS) secretion [1]. Exaggerated adrenarche and small size at birth are known to be related to polycystic ovary syndrome in girls, conveying an additional increase in the risk of metabolic syndrome [8,9].

Evidence suggests that postnatal growth pattern may also influence later metabolic and hormonal profile in children born SGA [1]. Actually, approximately 90% of infants born SGA catch up rapidly with their peers in terms of both weight and length during the first few months after birth. Thus, at 2 years of age, only 10% of children born SGA remain short; these are mainly the infants who were short at birth [10,11]. It has been postulated that the absence of catch-up growth early in infancy can be linked to endocrine disturbances [12]. During adolescence, the human body undergoes dramatic changes: increases in growth hormone (GH) and sex steroid secretion lead to increased insulin resistance and accelerated somatic growth, as well as to changes in body composition. All these processes may unmask underlying disturbances in metabolic function and hormonal profile. Although previous studies of adrenal function have looked at hormone changes in children born SGA, most have focused on children who remain short relative to their peers [13,14,15,16,17,18,19] or on SGA children undergoing investigations for precocious pubarche [20].

In this study, we aimed to investigate the relationship of adrenal function with postnatal growth and cardiovascular parameters in adolescents born SGA both with and without catch-up growth in comparison to their peers born appropriate for gestational age (AGA).

## 2. Materials and Methods

### 2.1. Statement of Ethics

The study was approved by the regional biomedical research ethics committee at the Lithuanian University of Health Sciences in Kaunas (Nr. BE-2-42, approved on 2011.06.14), and informed written consent was obtained from all parents prior to the study start.

### 2.2. Study Population

The study cohort included 102 Caucasian newborns (47 born SGA (24 boys/23 girls) and 55 born AGA [23 boys/32 girls]) born in Kaunas between 1998 and 2000 in whom growth was followed prospectively from birth at our center [21]. All children in the present study were born between 32 and 42 weeks of gestation. In children born SGA, birth weight and/or length was below 2 standard deviation scores (SDS) of the mean according to sex and gestational age, relative to Swedish birth weight and length references based on data from 800,000 healthy newborns born between 1990 and 1999 at a gestational age of 24–43 weeks [22]. Weight and length at birth in AGA newborns was between –2 and +2 SDS [22]. Children were examined at 2, 5, 12, 18 and 24 months after birth, and once during later childhood (mean age, 6.3 ± 0.07 years) [22,23]. At the time of investigation, children were between 11 and 14 years of age (mean age, 12.5 ± 0.1 years), and mean pubertal stages were: pubic hair development, 2 (interquartile range, 2–3), gonadarche (stages according to Tanner for breast development in girls and genital stages in boys), 2 (interquartile range, 2–3), testicular volume in boys, 5 mL (interquartile range, 2–9 mL). 40% of girls (*N* = 22) were post-menarche. As 7 of the 47 children born SGA did not experience catch-up growth in height up to 6 years of age (the last investigation before adolescence), the SGA group was analyzed as a whole, as well as according to whether they experienced catch-up growth in height or not.

### 2.3. Study Design

Anthropometric measurements: Height was measured to the nearest 0.1 cm using a Harpenden wall-mounted stadiometer (Holtain, Ltd., Crosswell, UK) and weight was measured to the nearest 0.1 kg using Soehnle electric scales (Soehnle, Backnang, Germany). Height and weight measurements were converted to SDS according to an algorithm for children from birth to 2 years [22], combined with an algorithm for total height and weight up to 20 years of age [24]. Body mass index (BMI) was calculated as a ratio of weight (kg) to height (m) and converted to SDS [25]. Pubertal stages were defined according to criteria of Tanner for breast development in girls, genital stages in boys and pubic hair development in both sexes. Testicular volume was measured using orchidometer.

**Figure 1:**
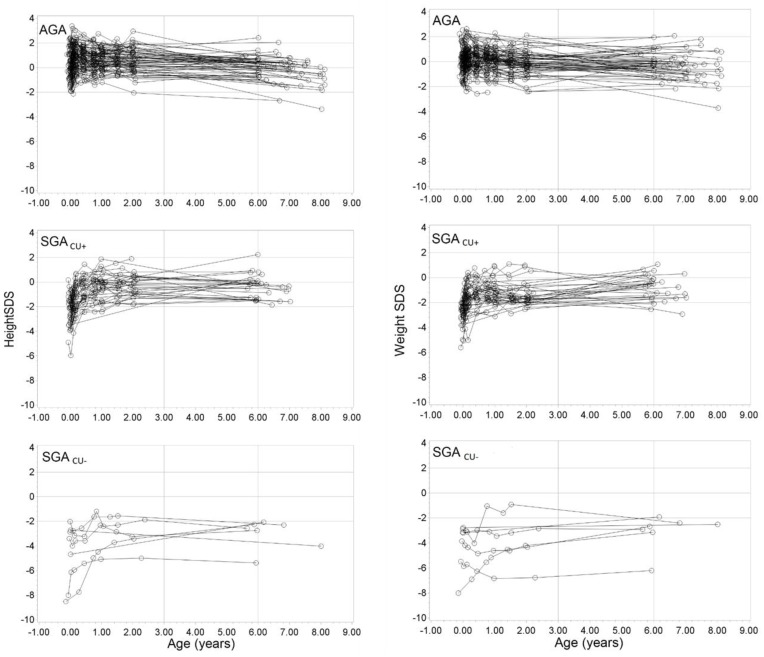
Change in height SDS and weight SDS during early childhood in children born appropriate for gestational age (AGA); children born SGA with catch-up growth (SGA_CU+_) and children born SGA without catch-up growth (SGA_CU−_).

Definition of catch-up growth: As height in adolescence is also influenced by onset of puberty, children born SGA were assigned to groups based on height before puberty: SGA children with a height above –2 SDS of the mean of the reference population according to sex and current age at 6 years of age were considered to be with catch-up growth (SGA_CU+_) [22,24]; SGA children with a height below –2 SDS of the mean at 6 years of age were considered to be without catch-up growth (SGA_CU−_). Height and weight growth during childhood are presented in Figure 1.

Skinfold thickness was measured to the nearest 0.1 cm at the subscapular, upper arm (triceps) and thigh area on the left-hand side using the Harpenden skinfold caliper. Two measurements were made at each site and the mean value used for analysis. Limb skinfold thickness was calculated as the sum of skinfold thicknesses in the upper arm and thigh area.

Cardiovascular parameters: Systolic and diastolic blood pressure and heart rate were measured using an automatic device with a cuff size appropriate for arm circumference. Two measurements were made of each variable and the mean value used for analysis.

### 2.4. Hormonal Measurements

Blood samples for hormone measurements were taken once between 08:00 and 09:00 h in the morning after overnight fasting, centrifuged immediately and stored at −20 °C until hormone concentrations were determined.

DHEAS concentration was determined using the commercially available DHEA-SO_4_ RIA reagents kit (Institute of Izotopes, Co., Ltd., Budapest, Hungary) which has a detection limit of 0.064 μmol/L, an intra-assay coefficient of variation (CV) of 4.6 and an inter-assay CV of 5.8%.

Cortisol concentration was determined using the Cortisol RIA reagent kit (DIAsource ImmunoAssays SA, Louvain, Belgium) with a detection limit of 2.5 nmol/L, an intra-assay CV of 7.7% and an inter-assay CV of 15.1%.

### 2.5. Statistical Analyses

The distribution of quantitative variables was tested for normality using the Kolmogorov–Smirnov test. Skewed parameters were log-transformed before analyses to ensure Gaussian distribution. Data for normally distributed variables are presented as mean and standard error of the mean (SEM); rank variables are presented as median and interquartile range. Study characteristics were compared using independent-sample t-tests for continuous variables and the χ^2^ test for binary and rank variables. Between-group comparisons for continuous variables were made using univariate general linear models with least square difference adjustment; all values were adjusted for current age, pubertal stage, BMI_SDS_ and sex; values for blood pressure analyses were additionally adjusted for current height. Partial correlation tests and Pearson correlation coefficients were used to analyze the relationship between DHEAS or cortisol levels and size at birth, early growth (in every measured interval) and cardiovascular parameters (blood pressure and heart rate).

Hierarchical multiple regression models using standardized coefficients were used to explain the variation in both DHEAS and cortisol levels, and to evaluate associations between DHEAS and cortisol levels during adolescence and perinatal and postnatal factors, after controlling for sex, age, pubertal stage, and BMI_SDS_. A *p* value of <0.05 was considered statistically significant. Statistical analyses were performed using SPSS statistical software (version 20.0, IBM^®^, New York, NY, USA).

## 3. Results

### 3.1. Study Population

At the current investigation, adolescents born SGA were significantly shorter and lighter than adolescents born AGA but had thicker subscapular skinfolds and higher waist to height ratio. There were no differences in current age and pubertal stages between total SGA and AGA groups. Study population characteristics are presented in Table 1. Birth weight_SDS_ was lower in boys born SGA compared to girls born SGA (*p* = 0.040). In adolescence, girls born AGA had thicker limb and subscapular skinfolds than boys born AGA (*p* = 0.011 and *p* = 0.016, respectively). There was no other difference in anthropometric measurement between boys and girls born either SGA or AGA.

In total, 7 of the 47 children born SGA (14.9%; 4 girls, 3 boys) did not experience catch-up growth and (their height was below −2 SDS at 6 years of age); the remaining 40 children born SGA experienced spontaneous catch-up growth. At the time of the study, SGA_CU−_ children were significantly shorter and lighter than SGA_CU+_ children and children born AGA (all *p* < 0.001). BMI_SDS_ was also lower in SGA_CU−_ children compared with SGA_CU+_ and AGA children (*p =* 0.003 and *p*=0.001, respectively); however, waist to height ratio was higher in SGA_CU−_ adolescents compared with SGA_CU+_ and AGA adolescents (*p* = 0.003 and *p* < 0.001, respectively). There were no differences in limbs or subscapular skinfold thicknesses between SGA_CU−_ and SGA_CU+_ adolescents (*p* = 0.456 and *p* = 0.719, respectively). At the time of study, SGA_CU+_ children were also shorter than children born AGA (*p* = 0.038), but there was no significant difference in weight_SDS_, BMI_SDS_ and waist to height ratio between SGA_CU+_ and AGA adolescents.

**Figure 2:**
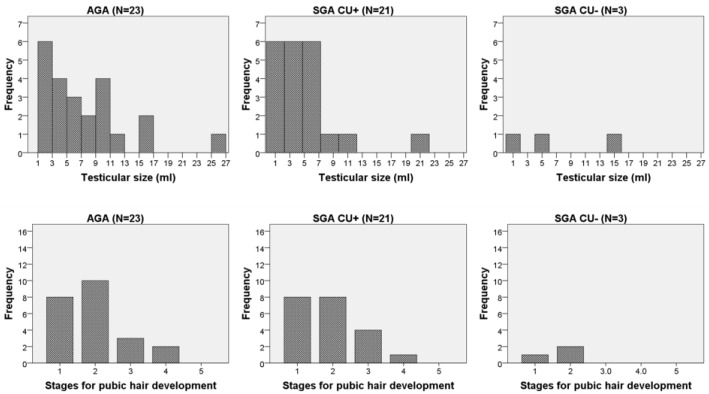
Testicular volume in adolescent boys according to whether they were born small for gestational age (SGA) or appropriate for gestational age (AGA). Boys born SGA are grouped according to catch-up growth (CU−, without catch-up growth; CU+, with catch-up growth).

**Figure 3:**
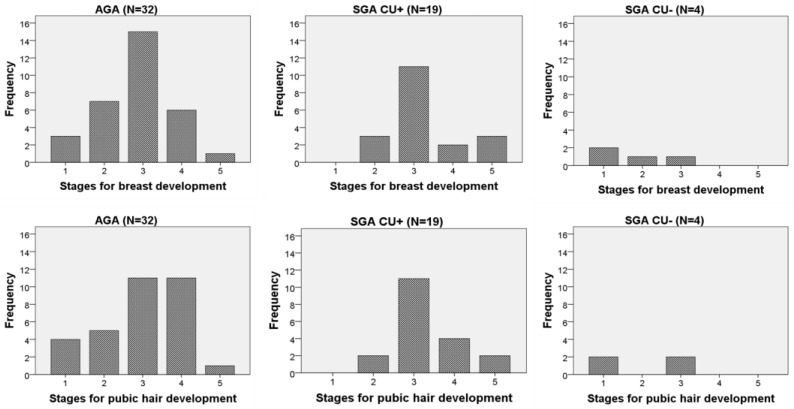
Stage of breast development according to Tanner in adolescent girls according to whether they were born small for gestational age (SGA) or appropriate for gestational age (AGA). Girls born SGA are grouped according to catch-up growth (CU−, without catch-up growth; CU+, with catch-up growth).

Pubic hair development stage did not differ between SGA and AGA groups in boys or girls (*p* = 0.820 and *p* = 0.964, Figure 2 and Figure 3, respectively).

As study children were in different pubertal stages, we compared pubertal maturation, expressed by testicular volume in boys and breast development stages in girls. When separated by sex, there was no difference in maturation between SGA and AGA children (all SGA vs. AGA, boys: *p* = 0.120; girls: *p* = 0.694). Also, there was no difference in testicular size between SGA_CU−_ and SGA_CU+_ boys (*p* = 0.481, Figure 2). The stages of breast development in SGA_CU+_ and AGA girls were similar (*p* = 0.179), with most girls being in the third stage for breast development according to Tanner (Figure 3). However, 2 out of 4 SGA_CU−_ girls were prepubertal (in the first stage for breast and pubic hair development) (*p* = 0.013) compared with SGA_CU+_ girls (0 out of 19); *p* = 0.024 compared with AGA girls (4 out of 31).

### 3.2. Factors Influencing Adrenal Hormone Levels

#### 3.2.1. DHEAS

##### Relationship to Size at Birth and Presence/Absence of Catch-Up Growth

Overall, DHEAS levels were significantly higher in children born SGA than in children born AGA (Table 2). Separate analysis of SGA_CU−_ and SGA_CU+_ groups showed that DHEAS levels were only significantly higher in SGA_CU+_ children compared with AGA children (Table 2). There was no significant difference between the two SGA groups (*p* = 0.963).

##### Relationship to Sex

When SGA and AGA children were separated into groups according to sex, there were no significant difference in DHEAS levels between boys born SGA compared with boys born AGA and no difference between the respective groups in girls (Table 2). Also, there was no difference in DHEAS levels between boys and girls born SGA or AGA (*p* = 0.557 and *p* = 0.412, respectively).

##### Relationship to Birth Characteristics

Overall, in the two SGA groups and the AGA group combined, DHEAS levels in adolescence were inversely associated with gestational age (*r* = –0.211, *p* = 0.034), birth weight (*r* = –0.306, *p* = 0.002), birth weight_SDS_ (*r* = –0.345, *p* < 0.001), birth length (*r* = –0.283, *p* = 0.004), birth length_SDS_ (*r* = –0.279, *p* = 0.005), BMI at birth (*r* = −0.296, *p* = 0.003) and ponderal index at birth (*r* = –0.229, *p* = 0.022) (analysis was adjusted for sex, current age, pubertal stage, and BMI_SDS_). Analyzing separately by sex, only in boys birth weight, length and BMI were associated with DHEAS levels in adolescence (*r* = −0.315, *p* = 0.035; *r* = −0.325, *p* = 0.029 and *r* = −0.299, *p* = 0.046, respectively) (Supplementary Materials Table S1).

##### Relationship to Postnatal Growth

For all children studied, when adjusted for sex, current age, pubertal stage, and BMI_SDS_, DHEAS levels were inversely related to weight gain during the first year of life (*r* = −0.289, *p* = 0.032) and increase in limb skinfold thickness during the second year of life (*r* = −0.416, *p* = 0.022), and directly associated with height velocity during the second year of life (*r* = 0.344, *p* = 0.032) and BMI gain during the first 6 years of life (*r* = 0.363, *p* = 0.003). DHEAS levels in adolescence were not related to current BMI_SDS_ (*r* = 0.161, *p* = 0.105; adjusted for sex, age, and pubertal stage). Analyzing separately by sex, only in girls weight gain during the first year of life was related to DHEAS levels in adolescence (*r* = −0.485, *p* = 0.012) (Supplementary Materials Table S1).

A multiple regression model was constructed to assess the relationship between variations in DHEAS levels and variables that were significantly related to DHEAS in the univariate analyses, but without significant inter-correlations, controlling for sex, age, pubertal stage, and current BMI_SDS_. In this model, measures of postnatal growth, but not birth size, were significantly related to DHEAS levels in adolescence (Table 3). The total variance explained by the model as a whole was 60.6%, *p* = 0.013. Postnatal growth explained 47.8% of the variance in DHEAS levels (*p* = 0.006).

#### 3.2.2. Cortisol

##### Relationship to Size at Birth and Catch-Up Growth

Overall, serum cortisol levels did not differ between the SGA and AGA groups. However, SGA_CU−_ children had significantly higher serum cortisol levels than both AGA (Table 4) and SGA_CU+_ children (*p* = 0.023 and *p* = 0.015, respectively).

##### Relationship to Sex

Overall, there were no sex differences in cortisol levels between the SGA and AGA groups (Table 4). Also, no differences were found between boys vs. girls in SGA or AGA groups (*p* = 0.985 and *p* = 0.779, respectively).

##### Relationship to Birth Characteristics

For all children studied, cortisol levels during adolescence did not correlate with size at birth or gestational age (Supplementary Materials Table S2).

##### Relationship to Postnatal Growth

Cortisol levels in adolescence correlated inversely with BMI gain during the second year of life and positively with increase in subscapular skinfold thickness between 2 years of age and adolescence (*r* = −0.306, *p* = 0.041 and *r* = 0.477, *p* = 0.002, respectively). In the SGA group, cortisol levels were positively associated with length growth velocity during the first 2 months of life (*r* = 0.470, *p* = 0.020), but inversely associated with length growth velocity between 2 and 5 months of life and with current weight_SDS_ (*r* = −0.511, *p* = 0.021 and *r* = –0.304, *p* = 0.048, respectively). Analyzing by sex, increase in subscapular skinfold thickness between 2 years of age was associated with cortisol levels only in adolescent girls (*r* = 0.584, *p* = 0.007) (Supplementary Materials Table S2).

Factors significantly related to cortisol levels at between 11 and 14 years of age and without significant inter-correlation were assessed in a multiple regression analysis. In this model, the most important factors related to cortisol level in adolescence were BMI gain during the second year of life and increase in subscapular skinfold thickness from 2 years to adolescence (Table 5). The total model explained 40.3% of the variance in cortisol levels at the time of investigation, *p* = 0.024. Postnatal growth (length growth velocity during the first 2 months of life, BMI gain during the second year of life and increase in subscapular skinfold thickness between 2 years of age and adolescence) explained 38.2% of the variance in cortisol levels (*p* = 0.002). Regression coefficients in the final model are presented in Table 4.

#### 3.2.3. Cardiovascular Parameters

Overall, there was no significant difference in systolic and diastolic blood pressure between the SGA and AGA groups. However, systolic blood pressure was higher in SGA_CU−_ children compared with SGA_CU+_ and AGA children (SGA_CU−_, 125.1 ± 5.4 mmHg vs. SGA_CU+_, 111.2 ± 1.9, *p* = 0.015; and vs. AGA, 109.0 ± 1.7 mmHg, *p* = 0.009). There was no difference in systolic blood pressure between SGA_CU+_ and AGA children (*p* = 0.426).

Diastolic blood pressure was significantly higher in SGA_CU−_ children compared with SGA_CU+_ and AGA children (SGA_CU−_, 76.9 ± 3.7 vs. SGA_CU+_, 66.5 ± 1.3 mmHg, *p* = 0.006; and vs. AGA, 66.0 ± 1.1 mmHg, *p* = 0.006). There was no difference in diastolic blood pressure between SGA_CU+_ and AGA children (*p* = 0.699).

Heart rate did not differ between SGA and AGA groups (81.3 ± 2.0 vs. 78.2 ± 1.9, *p* = 0.231, adjusted for sex, age, pubertal stage, and BMI_SDS_). Also, there were no significant difference between SGA_CU−_ and SGA_CU+_ adolescents (SGA_CU−_, 87.2 ± 5.8; SGA_CU+_, 80.5 ± 2.2; *p* = 0.269) or between AGA and SGA_CU−_ or SGA_CU+_ children (*p* = 0.127 and *p* = 0.340, respectively).

In the total cohort, systolic blood pressure was inversely related to weight gain between 1.5 and 6 years of life (*r* = −0.535, *p* = 0.003). Moreover, a positive relationship was established between cortisol levels and diastolic blood pressure (*r* = 0.203, *p* = 0.040).

## 4. Discussion

### 4.1. Principle Findings

In this study, we were able to demonstrate that both size at birth and early postnatal growth are important predictors of adrenal function in adolescence.

#### 4.1.1. DHEAS Association with Postnatal Growth

Previous studies have looked at DHEAS levels in preschool- and school-aged children who had been born SGA and most of them found higher levels in SGA children who had attained normal stature compared with those born AGA, but no difference between those who did not experience catch-up growth and AGA [13,14,15,16]. In combination with our data, these findings suggest that rapid postnatal growth—in particular during the second year of life—is an important predictor of adrenal androgen secretion in adolescence and it is more important than size at birth. Furthermore, together with the findings from Growth and Obesity Chilean Cohort Study in prepubertal children [26], our study suggests that not only length/height growth but also weight or BMI gain appears to be associated with adrenal function in later life.

A relationship between high BMI and hyper-responsiveness of the HPA axis has been shown in other recent studies [27]. In our study, however, a relationship between the increase in BMI rather than the actual BMI and DHEAS concentration in adolescence have been demonstrated: increase in BMI from birth to 6 years of life was found to be an independent predictor of the elevation of DHEAS concentration in adolescence, even after adjustment for current height, pubertal stage, and BMI_SDS_.

BMI can be seen as a surrogate marker of adiposity, although it does not reflect body adipose tissue compartmentalization. Indeed, the SGA-born children in our study were even leaner as adolescents than those born AGA, but had significantly higher waist to height ratios, indicating a more central distribution of adipose tissue. Furthermore, previous studies have found lean and total fat mass to be comparable in children born SGA or AGA [28,29], but with a tendency for increased visceral fat distribution in SGA children [28,30,31,32,33,34,35,36]. Additionally, DHEAS concentration in our study was inversely related to the increase in limb skinfold thickness, which reflects growth of peripheral adipose tissue. Taken together, it may be suggested that the specific postnatal growth pattern in SGA children, with rapid linear growth during the first 2 years of life, a greater gain in BMI from birth to 6 years of age, and a more central adipose tissue distribution in adolescence are associated with exaggerated adrenal androgen secretion.

#### 4.1.2. Secondary Sexual Characteristics and DHEAS Secretion

In the present study, the stages of pubic hair and gonadal development were similar in boys born SGA and those born AGA. Also, similarly to study by Beck Jensen [7], there were no differences in serum DHEAS levels between SGA and AGA boys. In previous studies higher DHEAS levels were described in girls with low birth weight and with a history of precocious pubarche [8]. In our study, there were no differences between the SGA and AGA groups in terms of DHEAS levels or stage of pubic hair and breast development. However, similarly to previous studies [37,38], girls born SGA were younger at onset of menarche than girls born AGA, suggesting that they experienced a faster transition through puberty. Interestingly, in regression analysis, current pubertal stage was not important predictor for DHEAS secretion, suggesting that early growth is a strongest predictor of variation of DHEAS secretion in adolescence.

#### 4.1.3. Cortisol Secretion and Cardiovascular Function

In our study, adolescents who were born SGA and did not experience catch-up growth had higher cortisol levels compared with adolescents born SGA with subsequent catch-up growth and AGA. Moreover, in children born SGA as a group, cortisol levels were significantly inversely associated with current weight_SDS_. Studies in adults have shown that cortisol secretion has a U-shaped relationship with BMI: higher cortisol secretion was linked not only to obesity, but also to being underweight [39]. These data are in line with our findings: SGA adolescents without catch-up growth had the highest cortisol levels and lowest BMI_SDS_. Furthermore, cortisol levels in adolescence were directly associated with the increase in subscapular skinfold thickness from 2 years of age to adolescence. The increase in subscapular skinfold thickness may reflect the tendency for a more centralized distribution of adipose tissue, which in turn may be related to a worse metabolic profile later in life.

Cortisol levels in our study were not related to pubertal stage, confirming findings from the study by Knutsson et al. which found a significant inter-individual variability in endogenous secretion pattern estimated as mean diurnal cortisol levels in a large group of healthy children. However, there was little within-individual variability in diurnal cortisol rhythm when the same individuals were followed longitudinally throughout puberty [40].

In a recent study by Kouda et al., adolescents with relatively low body fat but with a more centralized fat distribution than their peers, tended to have higher levels of blood pressure in later life [41]. In line with these data, in our study, SGA children without catch-up growth were leaner, had a greater waist to height ratio and higher systolic and diastolic blood pressure compared with children born SGA with subsequent catch-up growth or AGA. Previous studies have highlighted the increased risk of metabolic syndrome in children born SGA, particularly in those who experience catch-up growth [30,35,36]. In contrast, our data suggest that the risk of metabolic syndrome in later life may be higher in children born SGA who did not experienced catch-up growth than in those with catch-up growth. However, owing to the small number of SGA adolescents without catch-up growth in our study, these associations require confirmation in studies with larger sample sizes.

#### 4.1.4. Study Strengths and Limitations

The main strength of our study is the detailed longitudinal follow-up from birth to adolescence that was conducted within a single center, using reproducible and reliable measurement techniques. A further strength is the use of a single algorithm for height and weight from birth corrected for gestational age for all measurements.

Limitations of the study include the small sample size and different stages of pubertal development in study adolescents, although statistical analyses were adjusted for pubertal stages.

Assessment of adrenal function was also limited owing to the fact that samples were taken at a discrete time point, despite the well-known diurnal rhythm of adrenal hormones secretion; it is likely that assessment of the cortisol diurnal rhythm would be a more sensitive marker of adrenal function than a single measurement.

## 5. Conclusions

In conclusion, small size at birth is a predictor of higher DHEAS levels in adolescence in children born SGA who experienced catch-up growth. Children who were born SGA and did not experience catch-up growth are at risk of increased cortisol secretion and higher systolic and diastolic blood pressure in adolescence. Thus, the potential risk of metabolic consequences later in life in all individuals born SGA warrants further investigation.

## Figures and Tables

**Table 1:** Demographic and anthropometric characteristics of the study population (mean ± SEM, if not indicated otherwise).

	Total (*n* = 102)			SGA Subgroups				Boys (*n* = 47)			Girls (*n* = 55)		
	SGA (*n* = 47)	AGA (*n* = 55)	*P* Value	SGA_CU−_ (*n* = 7)	*P* Value	SGA_CU+_ (*n* = 40)	*P* Value	SGA (*n* = 24)	AGA (*n* = 23)	*P* Value	SGA (*n* = 23)	AGA (*n* = 32)	*P* Value
Gender, boys/girls (%)	51.1/48.9	41.8/58.2	0.353 *	42.9/57.1	0.969	52.5/47.5	0.400						
**At Birth**													
Gestational age (weeks)	38.7 ± 0.3	39.2 ± 0.2	0.134	38.7 ± 0.6	0.412	38.9 ± 0.2	0.340	38.5 ± 0.4	39.0 ± 0.4	0.385	39.0 ± 0.3	39.4 ± 0.2	0.274
Weight_SDS_	−3.09 ± 0.16	−0.08 ± 0.13	<0.001	−4.19 ± 0.73	<0.001	−2.89 ± 0.13	<0.001	−3.41 ± 0.27	0.06 ± 0.23	<0.001	−2.75 ± 0.16	−0.18 ± 0.15	<0.001
Length_SDS_	−2.62 ± 0.24	−0.07 ± 0.16	<0.001	−4.60 ± 1.0	<0.001	−2.27 ± 0.18	<0.001	−2.86 ± 0.34	−0.10 ± 0.26	<0.001	−2.37 ± 0.34	−0.04 ± 0.21	<0.001
Lean mass_SDS_	−1.82 ± 0.17	−0.04 ± 0.14	<0.001	−1.89 ± 0.86	<0.001	−1.80 ± 0.14	<0.001	−2.13 ± 0.23	0.18 ± 0.23	<0.001	−1.49 ± 0.24	−0.21 ± 0.17	<0.001
BMI (kg/m^2^)	11.1 ± 0.2	13.7 ± 0.2	<0.001	10.8 ± 0.7	<0.001	11.1 ± 0.16	<0.001	10.9 ± 0.2	14.0 ± 0.3	<0.001	11.3 ± 0.2	13.5 ± 0.2	<0.001
Ponderal index (kg/m^3^)	2.37 ± 0.03	2.70 ± 0.03	<0.001	2.52 ± 0.12	0.051	2.35 ± 0.03	<0.001	2.33 ± 0.03	2.75 ± 0.06	<0.001	2.42 ± 0.06	2.67 ± 0.04	0.001
**At the Time of Investigation**													
Age (years)	12.3 ± 0.1	12.6 ± 0.1	0.096	11.6 ± 0.34	0.056	12.4 ± 0.15	0.356	12.2 ± 0.2	12.4 ± 0.2	0.366	12.4 ± 0.2	12.7 ± 0.2	0.215
Height (cm)	152.1 ± 1.5	158.8 ± 1.0	<0.001	137.6 ± 4.4	<0.001	154.6 ± 1.2	0.016	151.3 ± 2.0	159.3 ± 1.8	0.005	152.9 ± 2.3	158.5 ± 1.2	0.023
Height_SDS_	−0.38 ± 0.19	0.36 ± 0.13	0.001	2.03 ± 0.67	<0.001	−0.10 ± 0.15	0.038	−0.34 ± 0.26	0.64 ± 0.18	0.004	−0.43 ± 0.7	0.16 ± 0.18	0.072
Weight (kg)	42.2 ± 1.4	49.3 ± 1.5	0.001	29.2 ± 2.7	<0.001	44.5 ± 1.3	0.021	41.9 ± 1.9	48.8 ± 1.8	0.012	42.5 ± 2.1	49.6 ± 2.2	0.029
Weight_SDS_	−0.36 ± 0.25	0.59 ± 0.21	0.004	−2.53 ± 0.67	<0.001	0.02 ± 0.23	0.074	−0.33 ± 0.36	0.73 ± 0.29	0.028	−0.40 ± 0.36	0.48 ± 0.29	0.062
BMI (kg/m^2^)	18.0 ± 0.4	19.4 ± 0.5	0.032	15.2 ± 0.6	0.011	18.5 ± 0.4	0.164	18.1±0.6	19.2 ± 0.6	0.209	17.9 ± 0.6	19.6 ± 0.7	0.094
BMI_SDS_	−0.17 ± 0.20	0.31 ± 0.17	0.069	−1.52 ± 0.49	0.001	0.06 ± 0.20	0.353	−0.05 ± 0.28	0.41 ± 0.24	0.212	−0.30 ± 0.29	0.23 ± 0.24	0.165
Subscapular skinfold thickness ***	11.1 ± 0.6	9.0 ± 0.5	0.004	13.1 ± 1.8	0.043	11.0 ± 0.7	0.041	8.8 ± 0.5	7.2 ± 0.5	0.098	13.2 ± 1.0	10.5 ± 0.8	0.051
Limb skinfold thickness ***	31.2 ± 1.3	28.1 ± 1.2	0.089	33.9 ± 3.9	0.138	30.8 ± 1.4	0.100	27.3 ± 1.6	24.9 ± 1.7	0.330	33.8 ± 2.1	31.5 ± 1.7	0.408
Waist circumference ***	66.1 ± 0.5	66.3 ± 0.4	0.912	66.8 ± 1.4	0.762	66.0 ± 0.5	0.786	66.6 ± 0.6	66.8 ± 0.6	0.957	66.1 ± 0.7	65.5 ± 0.6	0.510
Waist to height ratio ***	0.43 ± 0.01	0.42 ± 0.01	0.013	0.45 ± 0.01	<0.001	0.43 ± 0.01	0.071	0.43 ± 0.01	0.42 ± 0.01	0.009	0.43 ± 0.01	0.41 ± 0.01	0.060
Pubic hair **								2 [1,2]	2 [1,2]	0.820	3 [3,4]	3 [2,3,4]	0.964
Tanner stages of breast development in girls								-	-	-	3 [2,3]	3 [2,3]	0.694
Post-menarche (%)								-	-	-	27.3	50	0.158 *
Age at menarche (years)								-	-	-	12.0 ± 0.3	13.0 ± 0.3	0.048
Tanner stages of external genitalia development								1 [1,2,3]	2 [1,2,3]	0.204	-	-	-
Testicular volume (mL) **								4 [2,3,4,5,6]	6 [2,3,4,5,6,7,8,9,10]	0.120	-	-	-

AGA, appropriate for gestation age; BMI, body mass index; SGA, small for gestational age. SDS values for length, weight and BMI were calculated according to Swedish growth references [22,24,25]; * *P* value of χ^2^ test was used for comparison between SGA and AGA children. ** Data are presented as median and interquartile range. *** Data are presented as estimated mean and SEM, adjusted for sex, age, BMI_SDS_, and pubertal stage.

**Table 2:** Mean DHEAS levels (±SEM) in children born small for gestational age (SGA) or appropriate for gestational age (AGA): univariate general linear model, adjusted for sex, current age, pubertal stage, and BMI_SDS_.

	Group	*N*	SGA	*N*	AGA	*P* Value
**DHEAS (µmol/L)**	Total	47	4.65 ± 0.32	55	3.36 ± 0.29	0.007
	SGA_CU−_	7	4.45 ± 0.91			0.232
	SGA_CU+_	40	4.68 ± 0.34			0.008
	Boys	24	4.90 ± 0.51	23	3.54 ± 0.53	0.141
	Girls	23	4.22 ± 0.41	32	3.36 ± 0.35	0.112

BMI_SDS_, body mass index standard deviation score; SGA_CU−_, adolescents born SGA without catch-up growth; SGA_CU+_, adolescents born SGA with catch-up growth; DHEAS, dehydroepiandrosterone sulfate.

**Table 3:** Factors associated with dehydroepiandrosterone sulfate (DHEAS) levels in a multivariate linear regression model.

Variable	Standardized Coefficient β	B	95% CI for B	*P* Value
**Size at birth**				
Weight_SDS_	0.664	0.083	−0.048 to 0.214	0.200
Length_SDS_	−0.593	−0.057	−0.141 to 0.028	0.178
**Postnatal growth**				
0–6 yr. ∆ BMI (kg/m^2^)	0.657	0.069	0.011 to 0.128	0.022
1–2 yr. ∆ height (cm)	0.412	0.041	0.008 to 0.075	0.018
1–2 yr. ∆ limb skinfold thickness (mm)	−0.405	−0.022	−0.041 to −0.004	0.018
0–1 yr. ∆ weight (kg)	−0.270	−0.00006	−0.00014 to 0.00002	0.139
**Controlling factors**				
Current Age (y)	0.529	0.142	0.031 to 0.253	0.015
Sex	−0.429	−0.224	−0.412 to −0.037	0.021
Current pubertal stage	−0.163	−0.037	−0.135 to 0.060	0.433
Current BMI_SDS_	−0.093	−0.019	−0.111 to 0.074	0.682

BMI, body mass index. 1–2 yr. ∆ height: height velocity during second year of life. 0–6 yr. ∆ BMI: BMI gain during first 6 years of life. 1–2 yr. ∆ limb skinfold thickness: increase in limb skinfold thickness during second year of life. 0–1 yr. ∆ weight: weight gain during first year of life.

**Table 4:** Mean cortisol levels (±SEM) in children born small for gestational age (SGA) or appropriate for gestational age (AGA): univariate general linear model, adjusted for sex, current age, pubertal stage, and BMI_SDS_.

	Group	*N*	SGA	*N*	AGA	*P* Value
**Cortisol (nmol/L)**	Total	45	308.9 ± 17.4	55	301.9 ± 15.0	0.758
	SGA_CU−_	6	423.5 ± 49.2			0.023
	SGA_CU+_	39	294.0 ± 17.9			0.736
	Boys	24	318.8 ± 24.8	23	292.4 ± 26.9	0.567
	Girls	21	320.8 ± 23.1	32	296.6 ± 16.9	0.324

BMI_SDS_, body mass index standard deviation score; SGA_CU−_, adolescents born SGA without catch-up growth; SGA_CU+_, adolescents born SGA with catch-up growth.

**Table 5:** Multivariate linear regression model: associations between cortisol level and early growth.

Parameter	Standardized Coefficient β	B	95% CI for B	*P* Value
**Postnatal growth**				
2–12 yr. ∆ subscapular skinfold thickness (mm)	0.840	0.028	0.012 to 0.044	0.001
1–2 yr. ∆ BMI (kg/m^2^)	−0.459	−0.004	−0.007 to −0.001	0.010
0–2 mo. ∆ height (cm)	−0.160	−0.015	−0.043 to 0.014	0.311
**Controlling factors**				
Current BMI_SDS_	−0.596	−0.088	−0.158 to −0.017	0.016
Current age	0.169	0.034	−0.051 to 0.118	0.423
Sex	−0.245	−0.095	−0.232 to 0.042	0.167
Current pubertal stage	−0.136	−0.023	−0.098 to 0.052	0.538

1–2 yr. ∆ BMI—BMI gain between 1 and 2 years of life. 2–12 yr. ∆ subscapular skinfold thickness—increase in subscapular skinfold thickness from 2 years of life to adolescence. 0–2 mo. ∆ height: height velocity during first 2 months of life.

## Supplementary Materials

The following are available online at https://www.mdpi.com/1010-660X/55/5/167/s1, Table S1. Relationship between dehydroepiandrosterone sulfate (DHEAS) levels and size at birth, and early growth in the total group of adolescents, adjusted for current age, pubertal stage, and BMI_SDS_; Table S2. Relationship between cortisol levels and size at birth, and early growth in the total group of adolescents, adjusted for current age, pubertal stage, and BMI_SDS_.

## References

[1] Saenger P., Czernichow P., Hughes I., Reiter E.O. (2007). Small for gestational age: Short stature and beyond. Endocr. Rev..

[2] Clayton P.E., Cianfarani S., Czernichow P., Johannsson G., Rapaport R., Rogol A. (2007). Management of the child born small for gestational age through to adulthood: A consensus statement of the International Societies of Pediatric Endocrinology and the Growth Hormone Research Society. J. Clin. Endocrinol. Metab..

[3] Barker D.J., Winter P.D., Osmond C., Margetts B., Simmonds S.J. (1989). Weight in infancy and death from ischaemic heart disease. Lancet.

[4] Osterholm E.A., Hostinar C.E., Gunnar M.R. (2012). Alterations in stress responses of the hypothalamic-pituitary-adrenal axis in small for gestational age infants. Psychoneuroendocrinology.

[5] Liyanarachchi K., Ross R., Debono M. (2017). Human studies on hypothalamo-pituitary-adrenal (HPA) axis. Best Pract. Res. Clin. Endocrinol. Metab..

[6] Todorova B., Salonen M., Jaaskelainen J., Tapio A., Jaaskelainen T., Palvimo J., Turpeinen U., Hamalainen E., Rasanen M., Tenhola S. (2012). Adrenocortical hormonal activity in 20-year-old subjects born small or appropriate for gestational age. Horm. Res. Paediatr..

[7] Beck Jensen R., Vielwerth S., Larsen T., Hilsted L., Cohen A., Hougaard D.M., Jensen L.T., Greisen G., Juul A. (2011). Influence of fetal growth velocity and smallness at birth on adrenal function in adolescence. Horm. Res. Paediatr..

[8] Ibanez L., Potau N., Marcos M.V., De Zegher F. (2000). Adrenal hyperandrogenism in adolescent girls with a history of low birthweight and precocious pubarche. Clin. Endocrinol..

[9] Christodoulaki C., Trakakis E., Pergialiotis V., Panagopoulos P., Chrelias C., Kassanos D., Sioutis D., Papantoniou N., Xirofotos D. (2017). Dehydroepiandrosterone-sulfate, insulin resistance and ovarian volume estimation in patients with polycystic ovarian syndrome. J. Fam. Reprod. Health.

[10] Karlberg J., Albertsson-Wikland K. (1995). Growth in full-term small-for-gestational-age infants: From birth to final height. Pediatr. Res..

[11] Hokken-Koelega A.C., De Ridder M.A., Lemmen R.J., Den Hartog H., De Muinck Keizer-Schrama S.M., Drop S.L. (1995). Children born small for gestational age: Do they catch up?. Pediatr. Res..

[12] Albertsson-Wikland K., Boguszewski M., Karlberg J. (1998). Children born small-for-gestational age: Postnatal growth and hormonal status. Horm. Res..

[13] Francois I., de Zegher F. (1997). Adrenarche and fetal growth. Pediatr. Res..

[14] Ibanez L., Lopez-Bermejo A., Diaz M., Suarez L., de Zegher F. (2009). Low-birth weight children develop lower sex hormone binding globulin and higher dehydroepiandrosterone sulfate levels and aggravate their visceral adiposity and hypoadiponectinemia between six and eight years of age. J. Clin. Endocrinol. Metab..

[15] Dahlgren J., Boguszewski M., Rosberg S., Albertsson-Wikland K. (1998). Adrenal steroid hormones in short children born small for gestational age. Clin. Endocrinol..

[16] Boonstra V.H., Mulder P.G., de Jong F.H., Hokken-Koelega A.C. (2004). Serum dehydroepiandrosterone sulfate levels and pubarche in short children born small for gestational age before and during growth hormone treatment. J. Clin. Endocrinol. Metab..

[17] Radetti G., Renzullo L., Gottardi E., D’Addato G., Messner H. (2004). Altered thyroid and adrenal function in children born at term and preterm, small for gestational age. J. Clin. Endocrinol. Metab..

[18] Ong K.K., Potau N., Petry C.J., Jones R., Ness A.R., Honour J.W., de Zegher F., Ibanez L., Dunger D.B. (2004). Avon Longitudinal Study of Parents and Children Study Team. Opposing influences of prenatal and postnatal weight gain on adrenarche in normal boys and girls. J. Clin. Endocrinol. Metab..

[19] Ruys C.A., van der Voorn B., Lafeber H.N., van de Lagemaat M., Rotteveel J., Finken M.J.J. (2017). Birth weight and postnatal growth in preterm born children are associated with cortisol in early infancy, but not at age 8 years. Psychoneuroendocrinology.

[20] Eyzaguirre F.C., Bancalari R., Youlton R., Roman R., Silva R., Garcia H., Mericq V. (2009). Precocious pubarche: Experience in 173 cases. Rev. Med. Chil..

[21] Verkauskiene R., Albertsson Wikland K., Niklasson A. (2002). Variation in size at birth in infants born small for gestational age in Lithuania. Acta Paediatr..

[22] Niklasson A., Albertsson-Wikland K. (2008). Continuous growth reference from 24th week of gestation to 24 months by gender. BMC Pediatr..

[23] Valuniene M., Danylaite A., Kryziute D., Ramanauskaite G., Lasiene D.T., Lasas L., Verkauskiene R. (2009). Postnatal growth in children born small and appropriate for gestational age during the first years of life. Medicina.

[24] Wikland K.A., Luo Z.C., Niklasson A., Karlberg J. (2002). Swedish population-based longitudinal reference values from birth to 18 years of age for height, weight and head circumference. Acta Paediatr..

[25] Karlberg J., Luo Z.C., Albertsson-Wikland K. (2001). Body mass index reference values (mean and SD) for Swedish children. Acta Paediatr..

[26] Mericq V., Pereira A., Uauy R., Corvalan C. (2017). Early BMI Gain and Later Height Growth Predicts Higher DHEAS Concentrations in 7-Year-Old Chilean Children. Horm. Res. Paediatr..

[27] Incollingo Rodriguez A.C., Epel E.S., White M.L., Standen E.C., Seckl J.R., Tomiyama A.J. (2015). Hypothalamic-pituitary-adrenal axis dysregulation and cortisol activity in obesity: A systematic review. Psychoneuroendocrinology.

[28] Ibanez L., Lopez-Bermejo A., Suarez L., Marcos M.V., Diaz M., de Zegher F. (2008). Visceral adiposity without overweight in children born small for gestational age. J. Clin. Endocrinol. Metab..

[29] Ibanez L., Ong K., Dunger D.B., de Zegher F. (2006). Early development of adiposity and insulin resistance after catch-up weight gain in small-for-gestational-age children. J. Clin. Endocrinol. Metab..

[30] Ong K.K., Ahmed M.L., Emmett P.M., Preece M.A., Dunger D.B. (2000). Association between postnatal catch-up growth and obesity in childhood: Prospective cohort study. BMJ.

[31] Diaz M., Bassols J., Lopez-Bermejo A., de Zegher F., Ibanez L. (2015). Metformin treatment to reduce central adiposity after prenatal growth restraint: A placebo-controlled pilot study in prepubertal children. Pediatr. Diabetes.

[32] Sebastiani G., Diaz M., Bassols J., Aragones G., Lopez-Bermejo A., de Zegher F., Ibanez L. (2016). The sequence of prenatal growth restraint and post-natal catch-up growth leads to a thicker intima-media and more pre-peritoneal and hepatic fat by age 3–6 years. Pediatr. Obes..

[33] Alisi A., Panera N., Agostoni C., Nobili V. (2011). Intrauterine growth retardation and nonalcoholic Fatty liver disease in children. Int. J. Endocrinol..

[34] Tanaka Y., Kikuchi T., Nagasaki K., Hiura M., Ogawa Y., Uchiyama M. (2005). Lower birth weight and visceral fat accumulation are related to hyperinsulinemia and insulin resistance in obese Japanese children. Hypertens. Res..

[35] Biosca M., Rodríguez G., Ventura P. (2011). Central adiposity in children born small and large for gestational age. Nutr. Hosp..

[36] Eriksson M., Tynelius P., Rasmussen F. (2008). Associations of birthweight and infant growth with body composition at age 15—The COMPASS study. Paediatr. Perinat. Epidemiol..

[37] Ghirri P., Bernardini M., Vuerich M., Cuttano A.M., Coccoli L., Merusi I., Ciulli C., D’Accavio L., Bottone U., Boldrini A. (2001). Adrenarche, pubertal development, age at menarche and final height of full-term, born small for gestational age (SGA) girls. Gynecol. Endocrinol..

[38] Sloboda D.M., Hart R., Doherty D.A., Pennell C.E., Hickey M. (2007). Age at menarche: Influences of prenatal and postnatal growth. J. Clin. Endocrinol. Metab..

[39] Kumari M., Chandola T., Brunner E., Kivimaki M. (2010). A nonlinear relationship of generalized and central obesity with diurnal cortisol secretion in the Whitehall II study. J. Clin. Endocrinol. Metab..

[40] Knutsson U., Dahlgren J., Marcus C., Rosberg S., Bronnegard M., Stierna P., Albertsson-Wikland K. (1997). Circadian cortisol rhythms in healthy boys and girls: Relationship with age, growth, body composition, and pubertal development. J. Clin. Endocrinol. Metab..

[41] Kouda K., Ohara K., Fujita Y., Nakamura H., Iki M. (2016). Trunk-to-peripheral fat ratio predicts subsequent blood pressure levels in pubertal children with relatively low body fat- three-year follow-up study. Circ. J..

