# Supplementary material for: Adrenal Function in Adolescence is Related to Intrauterine and Postnatal Growth
Source: Medicina (Kaunas). 2019 May 20;55(5):167. doi: 10.3390/medicina55050167 (PMC6571974; doi:10.3390/medicina55050167)
Supplement: Supplementary file 1 [file medicina-55-00167-s001.pdf]

Table S1. Relationship between dehydroepiandrosterone sulfate (DHEAS) levels and size at birth, and early growth in the total group of adolescents, adjusted for current age, pubertal stage, and BMI_{SDS}.

	**Total Cohort**		**Boys**		**Girls**	
	**Correlation Coefficient r**	***P* Value**	**Correlation Coefficient *r***	***P* Value**	**Correlation Coefficient r**	***P* Value**
**Size at birth**						
Gestational age (weeks)	−0.211	0.034	−0.251	0.097	−0.117	0.415
Weight (kg)	−0.306	0.002	−0.315	0.035	−0.207	0.145
$Weight_{SDS}$	−0.345	<0.001	−0.274	0.069	−0.198	0.163
Length (cm)	−0.283	0.004	−0.325	0.029	−0.143	0.317
$Length_{SDS}$	−0.279	0.005	−0.247	0.101	−0.081	0.570
BMI (kg/m^2)	−0.296	0.003	−0.299	0.046	−0.250	0.077
Ponderal index	−0.229	0.022	−0.229	0.135	−0.212	0.135
**Postnatal growth**						
0–1 yr. Δ weight (kg)	−0.289	0.032	−0.173	0.389	−0.485	0.012
1–2 yr. Δ limb skinfold thickness (mm)	−0.416	0.022	−0.410	0.164	−0.508	0.053
1–2 yr. Δ height (cm)	0.344	0.032	0.427	0.087	0.316	0.175
0–6 yr. Δ BMI (kg/m^2)	0.363	0.003	0.380	0.051	0.308	0.067

BMI, body mass index. 0–1 yr. Δ weight: weight gain during first year of life. 1–2 yr. Δ limb skinfold thickness: increase in limb skinfold thickness during second year. 1–2 yr. Δ height: height velocity during second year of life. 0–6 yr. Δ BMI: BMI gain during first 6 years of life.

Table S2. Relationship between cortisol levels and size at birth, and early growth in the total group of adolescents, adjusted for current age, pubertal stage, and BMI_{SDS}.

	**Total Cohort**		Boys		Girls	
	**Correlation Coefficient *r***	***P* Value**	**Correlation Coefficient *r***	***P* Value**	**Correlation Coefficient *r***	***P* Value**
**Size at birth**						
Gestational age (weeks)	0.032	0.754	−0.108	0.482	0.182	0.211
Weight (kg)	−0.025	0.813	−0.125	0.413	0.094	0.521
$Weight_{SDS}$	0.004	0.968	−0.011	0.942	0.032	0.828
Length (cm)	−0.008	0.940	0.047	0.761	−0.064	0.662
$Length_{SDS}$	−0.011	0.914	0.133	0.385	−0.141	0.332
BMI (kg/m^2)	0.001	0.991	−0.166	0.275	0.202	0.163
Ponderal index	0.005	0.963	−0.270	0.077	0.262	0.069
**Postnatal growth**						
1–2 yr. Δ BMI (kg/m^2)	−0.306	0.041	−0.288	0.279	0.104	0.662
2–12 yr. Δ subscapular skinfold thickness (mm)	0.477	0.002	0.177	0.482	0.584	0.007
0–2 mo. Δ height (cm)	−0.076	0.551	−0.091	0.609	0.032	0.873
2–5 mo. Δ height (cm)	0.028	0.849	0.059	0.779	−0.049	0.833

BMI, body mass index. 1–2 yr. Δ BMI – BMI gain between 1 and 2 years of life. 2–12 yr. Δ subscapular skinfold thickness—increase in subscapular skinfold thickness from 2 years of life to adolescence. 0–2 mo. Δ height: height velocity during first 2 months of life. 2-5 mo. Δ height: height velocity between 2 and 5 months of life.
